# Supplementary material for: 3D Bioprinting of an Endothelialized Liver Lobule-like Construct as a Tumor-Scale Drug Screening Platform
Source: Micromachines (Basel). 2023 Apr 19;14(4):878. doi: 10.3390/mi14040878 (PMC10146619; doi:10.3390/mi14040878)
Supplement: Supplementary file 1 [file micromachines-14-00878-s001.zip › micromachines-2308537-supplementary.pdf]

Supplementary materials

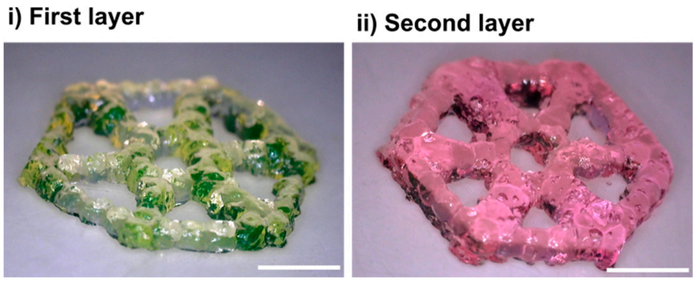

Figure S1. Bioprinting of heterogeneous lobule-like structures. i) one-layer lobule-like structure fabricated by printing GelMA hydrogel with green dye staining; ii) heterogeneous lobule-like structure fabricated by printing another type of hydrogel (gelatin) with red dye staining. Scar bar: 2 mm.

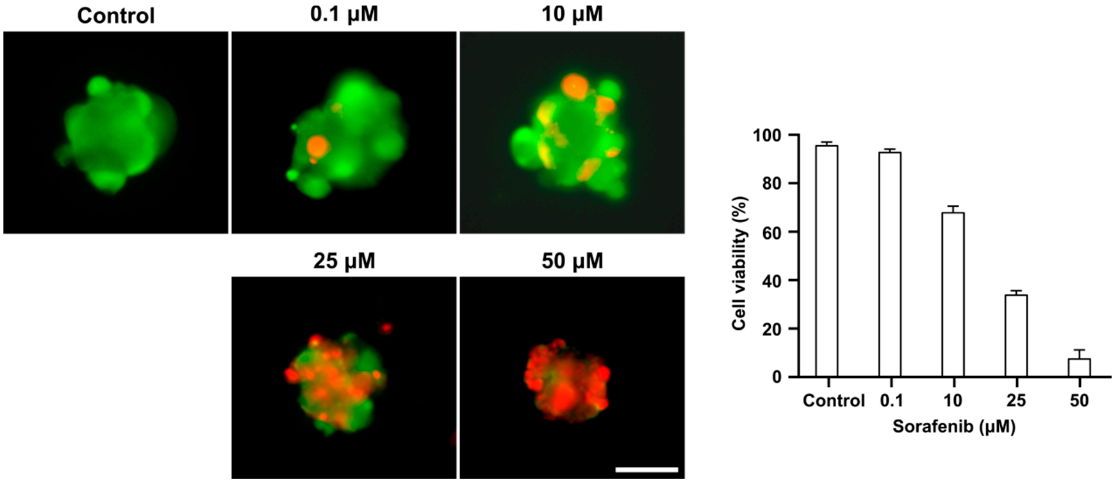

Figure S2. Drug screening of C3A spheroids. Live/dead staining of spheroids under Sorafenib treatment at different concentrations, and the corresponding cell viabilities were calculated. Scar bar: 200 μm.

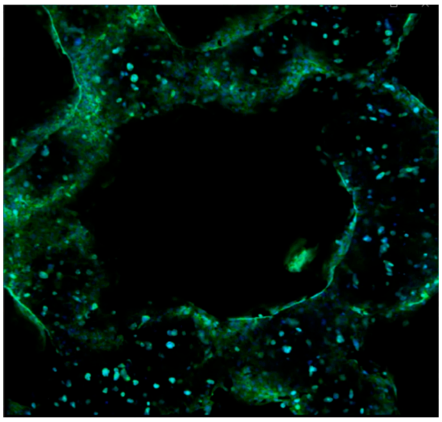

Video S1. Confocal image showing the endothelialized liver lobule-like construct from top to bottom.
